# Outcomes of external septorhinoplasty in a Turkish male population^☆^

**DOI:** 10.1016/j.bjorl.2017.04.010

**Published:** 2017-05-20

**Authors:** Gamze Didem Kilci, Engin Başer, Ayşegül Verim, Ömer Faruk Çalim, Bayram Veyseller, Orhan Özturan, Ahmet Altintaş, Mustafa Çelik

**Affiliations:** Bakirköy Dr. Sadi Konuk Training and Research Hospital, Istanbul, Turkey

**Keywords:** Septorhinoplasty, Ethnic facial harmony, Columellar incision type, Rhinobase program, Rinosseptoplastia, Harmonia facial étnica, Incisão columelar, Programa Rhinobase

## Abstract

**Introduction:**

The first and one of the most important steps in facial plastic surgery is accurate preoperative facial analysis and recording of data that may help the surgeon to check the outcomes of his/her techniques, promoting a surgeon's professional development.

**Objective:**

To evaluate the esthetic outcomes of external septorhinoplasty relevant to ethnic facial harmony and to investigate the relationship of the columellar incision scar with the type of skin and columellar incision type in a Turkish population.

**Methods:**

In total, 28 consecutive adult male patients with a mean age of 32.14 ± 10.66 years (range: 18–61 years) were included the study. Primary outcomes were preoperative and postoperative photogrammetric facial analyses of the patients including measurement of nasofrontal angle, nasolabial angle and nasal projection ratios (Gode) assessed according to the data derived from the Rhinobase program. Results were compared to facial proportions of the Turkish population. Columellar incision scar scores related to the Fitzpatrick skin type classification of the patients and columellar incision types used for the external approach were secondary outcomes of the study.

**Results:**

Mean preoperative and postoperative nasofrontal angles were 148.04° ± 8.18° and 144.50° ± 7.15°, respectively, while mean preoperative and postoperative nasolabial angles were 87.59° ± 14.01° and 98.50° ± 9.71°, respectively. Mean preoperative and postoperative nasal tip projection ratios were 0.56 ± 0.05 and 0.60 ± 0.06, respectively. The differences between pre- and postoperative measurements were all significantly different and were in accordance with Turkish nasal harmony. Columellar inverted “V” incisions were performed in 15 (53.6%) patients while “V” incisions were used in 13 (46.4%) patients. Fitzpatrick skin Type 4 was seen in 46.42% of the patients, Fitzpatrick Type 3 in 46.42% and Fitzpatrick Type 2 in 7.14% of the patients. No significant difference was seen between columellar scar scores according to skin type and columellar incision type used for external septorhinoplasty.

**Conclusions:**

This study demonstrated that outcomes for nasofrontal angle, nasolabial angle and nasal tip projection ratios analyzed using the Rhinobase program in patients who underwent external septorhinoplasty were similar to reference values for the Turkish population.

## Resumo

**Introdução:**

O primeiro e um dos mais importantes passos na cirurgia plástica facial é a análise pré-operatória facial precisa e registro de dados que podem ajudar o cirurgião a verificar os resultados de suas técnicas, promovendo seu desenvolvimento profissional.

**Objetivo:**

Avaliar os resultados estéticos da rinosseptoplastia externa relevantes para a harmonia étnica facial e investigar a associação da cicatriz de incisão columelar com o tipo de pele e tipo de incisão columelar em uma população turca.

**Método:**

No total, 28 pacientes adultos consecutivos com média de idade de 32,14 ± 10,66 anos (intervalo: 18-61 anos) foram incluídos no estudo. Os desfechos primários foram as análises faciais fotogramétricas pré-operatórias e pós-operatórias dos pacientes, incluindo a medida do ângulo nasofrontal, ângulo nasolabial e razões da projeção nasal (Gode), avaliados de acordo com os dados derivados do programa Rhinobase. Os resultados foram comparados às proporções faciais da população turca. Os escores de cicatriz de incisão columelar relacionados com a classificação de Fitzpatrick do tipo de pele dos pacientes e os tipos de incisão columelar usados para a abordagem externa foram os desfechos secundários do estudo.

**Resultados:**

Os ângulos nasofrontais pré- e pós-operatórios médios foram 148,04 ± 8,18° e 144,50 ± 7,15°, respectivamente, enquanto os ângulos nasolabiais pré- e pós-operatórios médios foram 87,59 ± 14,01° e 98,50 ± 9,71°, respectivamente. As razões médias da projeção nasal pré- e pós-operatória foram de 0,56 ± 0,05 e 0,60 ± 0,06, respectivamente. As diferenças entre as medidas pré- e pós-operatórias foram todas significativamente diferentes e estavam de acordo com a harmonia nasal turca. A incisão columelar em “V” invertido foi utilizada em 15 (53,6%) pacientes e a incisão em “V” foi utilizada em 13 (46,4%) pacientes. Pele Fitzpatrick tipo 4 foi observada em 46,42% dos pacientes, Fitzpatrick Tipo 3 em 46,42% e Fitzpatrick Tipo 2 em 7,14% dos pacientes. Não foi observada diferença significativa entre os escores de cicatriz columelar de acordo com o tipo de pele e o tipo de incisão columelar utilizados na rinosseptoplastia externa.

**Conclusões:**

Este estudo demonstrou que os desfechos para ângulo nasofrontal, ângulo nasolabial e razões de projeção nasal analisados pelo programa Rhinobase em pacientes submetidos à rinosseptoplastia externa foram semelhantes aos valores de referência para a população turca.

## Introduction

Apart from its major role in the respiratory mechanism, the nose is a component of the face that substantially contributes to facial esthetics. Therefore, septorhinoplasty (SRP), with ever-increasing interest, appears to be one the most commonly performed surgical techniques for esthetic and functional purposes. Regarding the incisional approach, SRP may be technically classified as either open (external) or closed rhinoplasty. Although, the technical and procedural aspects of these two approaches are similar, the external approach is preferred over the closed technique as it is more beneficial in terms of good anatomical exposure enabling easy learning and teaching for the rhinoplasty surgeon.^1^ However, undesirable scar formation and unpredictable poor healing of the columellar incision are the main drawbacks of this technique.

Pathologies underlying the nose, the patient's expectation for surgery, age, gender, race, facial harmony and her/his ethnic characteristics may present considerable variabilities between populations.^2^, ^3^, ^4^, ^5^, ^6^ Besides all of these factors which should be carefully examined, facial analysis including nasofrontal, nasolabial, nasomental angles, tip projection ratios and tip deviation angles should be performed preoperatively using certain objective measures and methods to obtain successful results.^7^, ^8^

Within this context, photographic techniques are preferred as commonly used methods in the preoperative planning and postoperative assessment of these landmarks.^7^, ^9^ Portrait photographs taken from six different directions are uploaded to various digital software programs developed for facial analysis and data drawn from these software are used in outcome assessment.

In this study, using the Rhinobase Borland Delphi software program, we aimed to critically examine pre- and postoperative facial analyses of patients who underwent open (external) approach SRP and to compare the esthetic results with the ethnic characteristics of the Turkish population.^10^ Columellar scar formation was also analyzed according to ethnic skin types and columellar incision type.

## Methods

This was a prospective, observational study conducted at the Otorhinolaryngology Department of our hospital between 2008 and 2011 with the approval of the local institutional ethics committee (Study ID B:30.2.BAV.0.05.05/31). All volunteers were provided with information about the procedures, and written informed consent was obtained before the study. Twenty-eight consecutive adult male patients who underwent primary external SRP with a diagnosis of septonasal deformity were included in the study.

Patients were excluded if they had a history of previous SRP, additional sinonasal pathologies (chronic rhinosinusitis with or without polyposis), and female patients were also excluded to minimize variability because of gender differences.

Demographic information, anamnesis, previous medication, systemic diseases, detailed endoscopic examination and Fitzpatrick skin type classification of the patients were entered into the hospital database.^11^, ^12^

**Figure 1:**
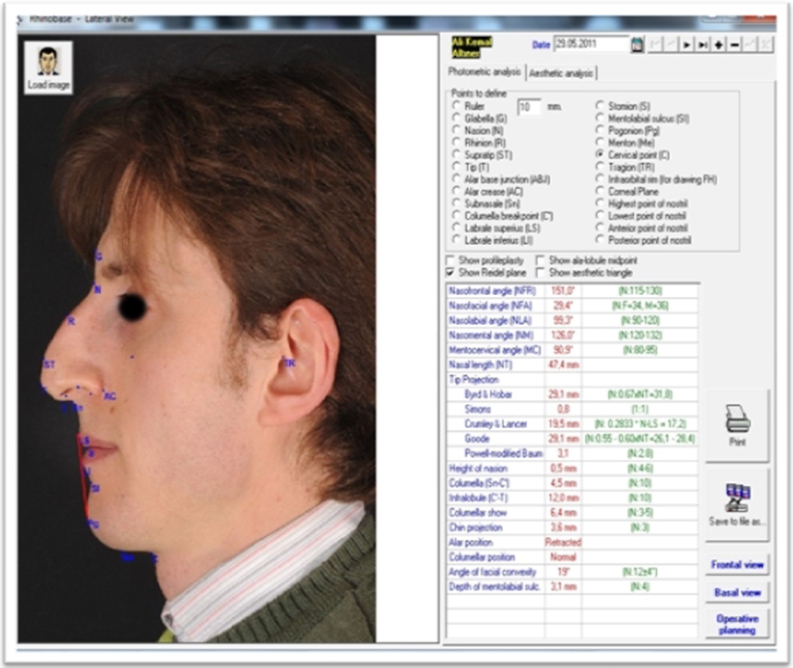
Preoperative lateral facial analysis.

**Figure 2:**
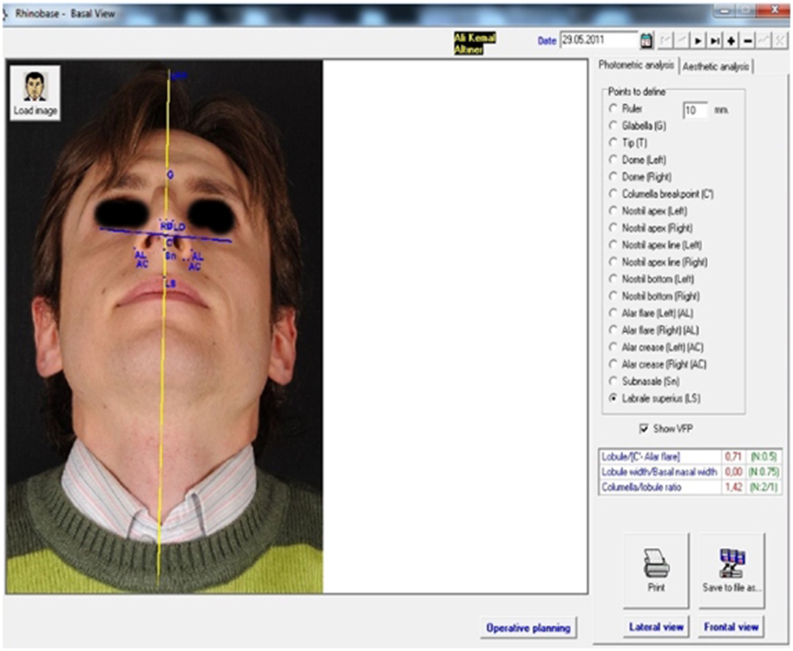
Preoperative basal facial analysis.

Portrait photographs from the anterior, basal, right lateral, left lateral, right oblique and left oblique views (six directions) were taken preoperatively by a professional expert in rhinoplasty photography and were uploaded to the Rhinobase Borland Delphi software program (version 4.0 for Windows; Inprise Corp, Scotts Valley, CA, USA).^10^ Anatomical landmarks (tip, supratip, subnasale, nasion, rhinion, etc.) were marked on the photographs and in the appropriate box seen on the right side of the screen. Measurements of corresponding lengths, heights, distances, ratios (tip projection ratio) and angles (Nasofrontal Angle (NFA), Nasolabial Angle (NLA), etc.), automatically calculated in the Rhinobase program, were displayed on the screen and stored in the program (Figure 1, Figure 2).

### Surgery and postoperative care

External SRP under general anesthesia was preferred for the procedure. Patients were divided into either columellar inverted “V” or “V” incision groups using a coin toss. Incisions were closed using 4/0 absorbable polyglactin for the subdermal layer, and 5/0 nonabsorbable polypropylene for the skin layer. Topical antibiotic ointment was applied to the sutures until their removal on postoperative day 5.

### Follow-up assessments and outcome measures

**Figure 3:**
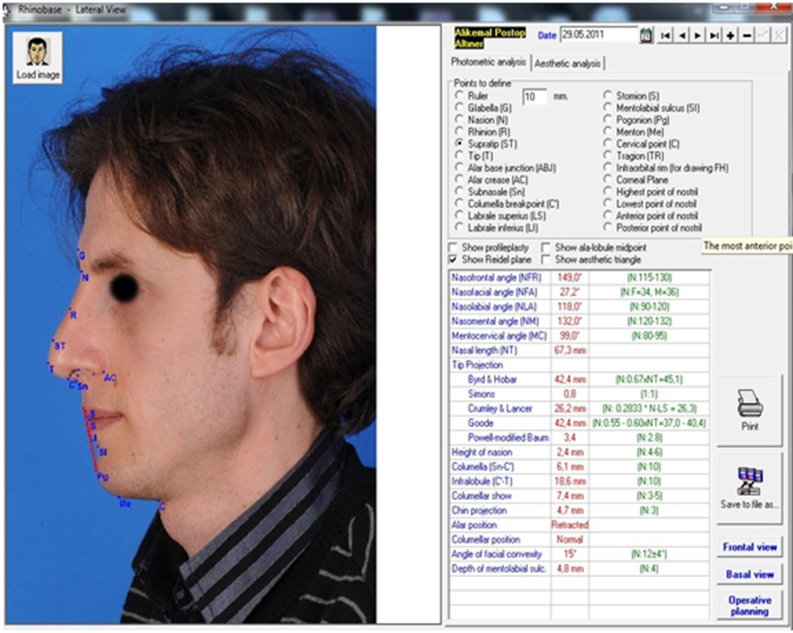
Postoperative lateral facial analysis (sixth month).

**Figure 4:**
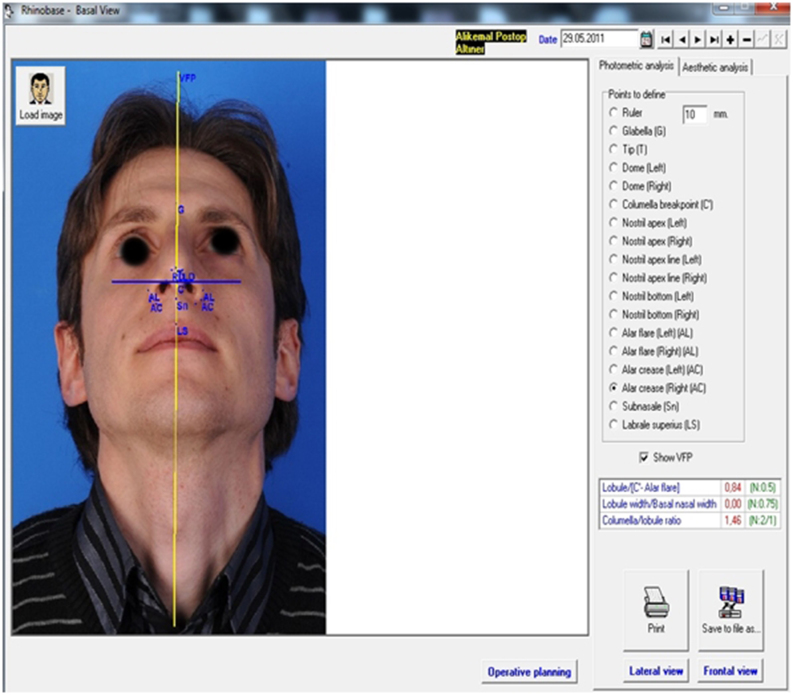
Postoperative basal facial analysis (sixth month).

Patients were all followed up for a mean of 9.82 ± 6.15 months (range 6–30 months) after surgery. Similar to the procedure for preoperative photogrammetric measurements with the Rhinobase software program, patients’ portrait photographs were all reuploaded and NLA, NFA and nasal tip projection ratios were reevaluated at postoperative follow-up (Figure 3, Figure 4). Photogrammetric facial analyses were accepted as the primary outcome measures of the present study.

Secondary outcome measures were columellar scar assessments based on the Stony Brook Scar Evaluation Scale (SBSES) modified by Verim et al. for use with columellar scars.^13^, ^14^ The presence or absence of scars of width >2 mm, elevation or depression, discoloration, notching, and overall appearance were assigned 0 or 1 point for each of these items. Total scores were categorized into five groups ranging from 0 (worst), 1 (poor), 2 (mild), 3 (moderate), 4 (good) to 5 (best – no scar). Columellar scars were evaluated with regard to Fitzpatrick skin type classification of the patients and columellar incision type used in the SRP.

### Statistical analysis

NCSS (Number Cruncher Statistical System) 2007 and PASS (Power Analysis and Sample Size) 2008 Statistical Software (NCSS LLC, Kaysville, UT, USA) has been used for statistical analysis of the results. Descriptive statistics (mean, standard deviation, frequency, median) were used in the evaluation of the study data. Data were compared using the Chi-squared test and Paired Sample *t*-test. Statistical significance was accepted at *p* < 0.05 with *p* < 0.01 being very significant.

## Results

Twenty-eight consecutive adult male patients with a mean age of 32.14 ± 10.66 years (range: 18–61 years) completed the study. Postoperative follow-up ranged between 6 and 30 months with a mean of 9.82 ± 6.15 months. The columellar inverted “V” incision was performed in 15 (53.6%) patients while the columellar “V” incision was used in 13 (46.4%) patients.

The Fitzpatrick skin type classification of the patients was as follows: 2 (7.1%) patients had Type 2, 13 (46.4%) patients Type 3, and 13 (46.4%) patients Type 4. Demographics and Fitzpatrick skin classification of the patients are detailed in Table 1.

**Table 1:** Distribution of patients’ age, follow-up period, Fitzpatrick skin types, and columellar incision types.

	Min-Max	Mean ± SD
*Age (years)*	18–61	32.14 ± 10.66
*Postoperative follow-up (months)*	6–30	9.82 ± 6.15

	Number of patients	%
*Fitzpatrick skin types*		
Type 2	2	7.1
Type 3	13	46.4
Type 4	13	46.4
*Columellar incision types*		
Inverted V	15	53.6
V	13	46.4

Mean preoperative NFA, NLA and tip projection ratios of the patients retrieved from the Rhinobase program were respectively 148.04° ± 8.18°, 87.59° ± 14.01°, and 0.56 ± 0.05. However, mean postoperative NFA, NLA and tip projection ratios were 144.50° ± 7.15°, 98.50° ± 9.71°, and 0.60 ± 0.06, respectively. Mean NFA, NLA and tip projection ratios all improved significantly after SRP (Paired Sample *t*-test; *p* < 0.01; *p* = 0.001, 0.001, and 0.003, respectively). Detailed analyses of NFA, NLA and tip projection ratios are displayed in Table 2.

**Table 2:** Preoperative, postoperative nasofrontal angle, nasolabial angle, and tip projection ratios of the patients.

	Mean ± SD	*p*-Value
*Nasofrontal angle (NFA; degrees)*		
Preoperative	148.04 ± 8.18	0.001^a^
Postoperative	144.50 ± 7.15	
*Nasolabial angle (NLA; degrees)*		
Preoperative	87.59 ± 14.01	0.001^a^
Postoperative	98.50 ± 9.71	
*Tip projection ratios*		
Preoperative	0.56 ± 0.05	0.003^a^
Postoperative	0.60 ± 0.06	

Paired sample *t*-test.

a *p* < 0.01.

Columellar scar assessments at long-term follow-up demonstrated that 8 (28.6%) patients had a columellar scar depressed in relation to the surrounding skin; 3 (10.7%) patients had a scar darker than the surrounding skin; 5 (17.9%) patients had notching; 2 (7.1%) had a scar with poor overall appearance; 1 (3.6%) patient had a scar wider than 2 mm. Columellar scar assessments according to Stony Brook Scar Evaluation Scores disclosed 2 (7.1%) patients with a poor (1/5) columellar scar, 3 (10.7%) patients with a mild (2/5) columellar scar, 4 (14.3%) patients with a moderate (3/5) scar, and 19 (67.9%) patients without scar formation (5/5). Patients’ columellar scars classified according to Stony Brook Scar Evaluation Scores are presented in Table 3.

**Table 3:** Distribution of scar evaluation parameters and scores of the patients.

Stony Brook Scar Evaluation	Number of patients	%
*Depressed compared with surrounding skin*	8	28.6
*Darker than the surrounding skin*	3	10.7
*Notching*	5	17.9
*Poor overall appearance*	2	7.1
*Width ≥2* *mm*	1	3.6
*Distribution of Scar Scores*		
Poor (1/5)	2	7.1
Mild (2/5)	3	10.7
Moderate (3/5)	4	14.3
No Scar (5/5)	19	67.9

Evaluation of Stony Brook scar scores in relation to Fitzpatrick skin type classification demonstrated no statistically significant difference between scar scores of the patients and Fitzpatrick Skin Type 2, Type 3, or Type 4 (Chi-Square test; *p* > 0.05) (Table 4). Likewise, evaluation of Stony Brook scar scores in relation to columellar incision type used for external approach SRP (Inverted “V” vs. “V” incision) demonstrated no statistically significant difference between scar scores of the patients and type of columellar incision employed for external SRP (Table 5).

**Table 4:** Evaluation of Stony Brook Scar Scores according to Fitzpatrick skin type.

Stony Brook Scar Scores	Fitzpatrick skin type			*p-*Value
	Type 2	Type 3	Type 4	
	*n* (%)	*n* (%)	*n* (%)	
1/5 (poor)	0 (0)	2 (15.4)	0 (0)	0.587
2/5 (mild)	0 (0)	1 (7.7)	2 (15.4)	
3/5 (moderate)	0 (0)	1 (7.7)	3 (23.1)	
5/5 (no scar)	2 (100)	9 (69.2)	8 (61.5)	

Chi-Squared test. *p* < 0.05.

**Table 5:** Evaluation of Stony Brook Scar Scores according to columellar incision type.

Stony Brook Scar Scores	Columellar incision type		*p*-Value
	Inverted V incision	V incision	
	*n* (%)	*n* (%)	
1/5 (poor)	2 (13.3)	0 (0)	0.066
2/5 (mild)	3 (20)	0 (0)	
3/5 (moderate)	3 (20)	1 (7.7)	
5/5 (no scar)	7 (46.7)	12 (92.3)	

Chi-Square test; *p* < 0.05.

Therefore, skin type (Fitzpatrick Type 2, Type 3, or Type 4) and columellar incision type used (inverted “V” or “V” incision) were not factors influencing scar formation after external SRP.

## Discussion

The first and one of the most important steps in facial plastic surgery is accurate preoperative facial analysis and recording of data that may help the surgeon to check the outcomes of his/her techniques, promoting a surgeon's professional development.^7^, ^15^, ^16^

Photogrammetric facial analysis, a method commonly used in facial analysis, enables objective substantiation and archiving of the outcomes of SRP.^7^, ^9^ This method is more reliable than cephalometric analysis in soft tissue profile analysis, and for determining the racial and ethnic differences in normal facial profiles. Moreover, angle and ratio measurements, which are independent of image dimensions, are the major advantages of this method over cephalometric analysis.^9^, ^17^

Various soft tissue facial analysis programs based on 2D or 3D photographic documentation have been reported in the literature.^18^, ^19^, ^20^ From these, Rhinobase, a free program with an automated photographic analysis tool achieving complete facial analysis in less than 15 min, is our preferred program for pre- and postoperative analyses.^10^

Several studies have reported that ethnicity, race, and gender are factors determining facial ratios and angles in populations.^7^, ^9^, ^16^, ^21^, ^22^ However, although endorsed in the study by Biller and Kim in 2009, ethnicity and age were pointed out to be of secondary importance to the evaluation of individual facial harmony.^23^

Knowledge of facial esthetic measurements of patients in a particular population is a prerequisite for precise facial analysis.^24^ The nasion, one of the conspicuous landmarks in facial harmony, and the angle derived from this reference point (nasofrontal angle) should be carefully considered in lateral profiles in an attempt to gain measurements specific to the ethnicity of the patient.

In 2011, in a study by Gode et al. in Turkey comprising 40 controls and 40 patients who were to undergo SRP, mean NFA measurements determined after soft tissue facial analysis were about 143.3° ± 8.3° in controls who were pleased with their facial appearance. The authors stated no significant differences with regard to patients’ gender.^7^

In 2008, standard photogrammetric facial analysis of another Turkish population including 111 controls revealed NFA measurements of males (mean ± SD 139.5° ± 11°) to be unrelated to gender. However, when considering the very large range of NFAs, the authors concluded that NFA measurements varied substantially between Turks.^25^ In a study covering 100 Turks in 2009, Malkoç et al. also reported mean male NFAs (146° ± 8.19°) to be unrelated to gender.^26^

Mean nasofrontal angles of our patients were 148.04° ± 8.18° preoperatively and 144.50° ± 7.15° in postoperative follow-up. The decrease in the mean measurement of nasofrontal angles was very significant (*p* = 0.001). Moreover, mean postoperative NFAs of our patients were within the range of mean NFAs, varying between 139.5° ± 11° and 146° ± 8.19°, in Turkish people who were satisfied with the appearance of their nose.^7^, ^25^, ^26^

In a recent study evaluating mean nasal anthropometric measurements in young Turkish males in 2006, Turks living on the Black Sea coast were found to have a NFA (134.96° ± 7.7°) more acute than in our cohort.^22^ Most likely, this diversity originates from facial structures differing from one region to another in Turkey. In our opinion, heterogeneity of the population should be borne in mind before interpreting average facial analysis measurements. Indeed, the demographic composition of Anatolian Territory presents considerable variations as a result of being occupied by multiple immigrations in the past.

Nasal tip contouring has always been a critical factor in achieving successful rhinoplasty.^27^ Projection, rotation, shape, and soft tissue thickness are the main characteristics of an ideal nasal tip.^28^ Rhinoplasty is known to require elaboration of tip projection and rotation as key components of surgical success.^29^

In an effort to identify the improvement in tip projection, preoperative and postoperative tip projection ratios were compared in our study. The very significant increase that we identified after surgery (0.56 ± 0.05 vs. 0.60 ± 0.06; *p* < 0.003) was consistent with tip projection ratios (0.55–0.60 of the distance from the nasion to the nasal tip) defined by Goode.^30^

In our study, NLA was selected to be the third parameter for analyzing the Turkish male profile before and after SRP. As mentioned before, when considering NFA and tip projection ratios, NLA has proved to differ among various ethnicities and races.^22^, ^25^, ^26^ Similar to the outcomes for NFA and tip projection ratios, preoperative and postoperative NLA values were very significantly improved in our patients (87.59° ± 14.01° vs. 98.50° ± 9.71°; *p* = 0.001). Furthermore, NLA measurements of our cohort were in keeping with the mean NLAs of the Turkish population reported by Kale-Varlık^25^ and Malkoc et al.^26^ (98° ± 13.7° and 101° ± 10°, respectively).

Along with the primary outcome parameters used for profile analysis in SRP, columellar incision scars were included in the scope of secondary outcomes in the present study. The effects of incision type used in the external approach and pigmentation of the skin were investigated on columellar scar outcome assessed using the Stony Brook Scar Evaluation Scale.^13^ In the present study, 7.1% of patients had poor scar formation, 10.7% had mild scar formation and 68% of patients had no columellar scar. In summary, our failure rate in columellar scar formation was about 7.1%. These results were poorer than for some authors whose scar rate did not exceed 2%.^31^, ^32^ However, when compared to the columellar scar evaluation of Bafaqeeh and Al-Qattan in an Arabian population, our results were seen to be better than those reported by these authors who attributed the high rate of scar formation to the thick and dark skin of their patients.^33^

In contrast to the assertions of Bafaqeeh and Al-Qattan, we were not able to confirm a relationship between poor scar formation and high pigment concentration of the skin. In fact, we preferred the widely used Fitzpatrick skin type classification for examining the relationship between scar formation and skin tone, and found no correlation between these two parameters. From this point, our study was rather in line with the opinions of Adamson who considered the columella to be a preferred site for healing, even in darker skins.^34^

The lack of correlation between columellar scar formation and columellar incision type is the final point of our study. Controversy exists on the relationship between scar formation and the type of columellar incision used in external SRP. There are some who suggest that, with the inverted “V” incision, there may be a more satisfactory scar in terms of visibility. However, some do not agree, based on the opinion that finesse should be required for suturing incision lines.^31^, ^32^, ^35^, ^36^

Our observations did not confirm the superiority of a particular incision type over another in terms of preventing poor healing. Indeed, 53.6% of our patients were operated using an inverted “V” incision and 46.4% with a “V” incision and no correlation was found between columellar scar formation and incision technique employed.

Although we achieved interesting results, there are limitations to our study. The size of the study population may also be a limitation. We could not asserted that columellar scar formation was not related to the type of columellar incision used or to pigment concentration of the skin because of the small study population. The other limitations of the present study are that the limited number of parameters analyzed. However, considering the differences in NLA, NFA and tip projection ratios between genders, we preferred to study male patients to avoid any possible bias. Furthermore, this study was rather built on the pre- and postoperative profile analysis of a particular population. Further studies involving larger numbers of patients are needed to confirm these initial findings.

## Conclusion

In conclusion, the results of the present study demonstrated that the mean NFA, NLA and tip projection ratios of Turkish men who required improvement in their nasal appearance and symptoms were, respectively, 148.04° ± 8.18°, 87.59° ± 14.01° and 0.56 ± 0.05, whereas postoperative evaluation of the same parameters used in profile analysis yielded 144.50° ± 7.15°, 98.50° ± 9.71° and 0.60 ± 0.06. Postoperative profile measurements were in line with those of Turkish men who were pleased with the appearance of their nose: 7.1% of patients healed with a poor columellar scar, while 68% healed with no scar.

## Conflicts of interest

The authors declare no conflicts of interest.
